# Prediction of in-hospital death following acute type A aortic dissection

**DOI:** 10.3389/fpubh.2023.1143160

**Published:** 2023-03-29

**Authors:** Junquan Chen, Yunpeng Bai, Hong Liu, Mingzhen Qin, Zhigang Guo

**Affiliations:** ^1^Clinical School of Thoracic, Tianjin Medical University, Tianjin, China; ^2^Department of Cardiovascular Surgery, Tianjin Chest Hospital, Tianjin, China; ^3^Department of Cardiovascular Surgery, First Hospital of Nanjing Medical University, Nanjing, China

**Keywords:** in-hospital death, risk prediction, acute type A aortic dissection, prediction model, biomarker

## Abstract

**Background:**

Our goal was to create a prediction model for in-hospital death in Chinese patients with acute type A aortic dissection (ATAAD).

**Methods:**

A retrospective derivation cohort was made up of 340 patients with ATAAD from Tianjin, and the retrospective validation cohort was made up of 153 patients with ATAAD from Nanjing. For variable selection, we used least absolute shrinkage and selection operator analysis, and for risk scoring, we used logistic regression coefficients. We categorized the patients into low-, middle-, and high-risk groups and looked into the correlation with in-hospital fatalities. We established a risk classifier based on independent baseline data using a multivariable logistic model. The prediction performance was determined based on the receiver operating characteristic curve (ROC). Individualized clinical decision-making was conducted by weighing the net benefit in each patient by decision curve analysis (DCA).

**Results:**

We created a risk prediction model using risk scores weighted by five preoperatively chosen variables [AUC: 0.7039 (95% CI, 0.643–0.765)]: serum creatinine (Scr), D-dimer, white blood cell (WBC) count, coronary heart disease (CHD), and blood urea nitrogen (BUN). Following that, we categorized the cohort's patients as low-, intermediate-, and high-risk groups. The intermediate- and high-risk groups significantly increased hospital death rates compared to the low-risk group [adjusted OR: 3.973 (95% CI, 1.496–10.552), *P* < 0.01; 8.280 (95% CI, 3.054–22.448), *P* < 0.01, respectively). The risk score classifier exhibited better prediction ability than the triple-risk categories classifier [AUC: 0.7039 (95% CI, 0.6425–0.7652) vs. 0.6605 (95% CI, 0.6013–0.7197); *P* = 0.0022]. The DCA showed relatively good performance for the model in terms of clinical application if the threshold probability in the clinical decision was more than 10%.

**Conclusion:**

A risk classifier is an effective strategy for predicting in-hospital death in patients with ATAAD, but it might be affected by the small number of participants.

## Graphical Abstract

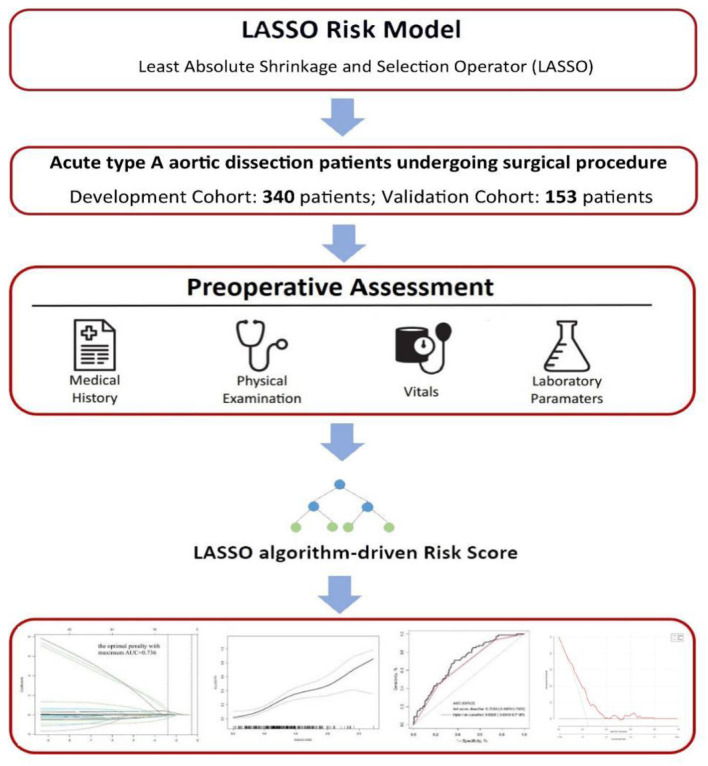

## Introduction

Acute type A aortic dissection (ATAAD) is a cardiovascular emergency that poses a serious risk to life and has a greater rate of short-term morbidity and mortality (1). According to estimates, there is a 1%−2% probability of death every hour, and non-operative therapy caused mortality in ~60% of patients (2, 3). The rate of mortality for patients with ATAAD continued to be as high as 22% according to the latest research from the International Registry of Acute Aortic Dissection, which comprised 4,428 individuals from 1995 to 2013 (4).

Acute type A aortic dissection is frequently managed by medical therapy and immediate surgery during the acute stage. The current standard of care for patients with ATAAD is still surgical repair, which has been found to be the most effective therapy, with a mortality rate of 27% compared to 56% for those managed medically in-hospital (5). Despite improvements in perioperative care and surgical procedures, mortality rates remain high (4, 6). Therefore, it is crucial to identify patients with high-risk ATAAD (7).

Although the fact that numerous models have been established to foresee morbidity or mortality in heart surgery (8, 9), there is no golden standard for the prognosis of ATAAD, and there are no predictive models that only use preoperative factors to predict in-hospital death risk after the surgical management of ATAAD.

In order to promote clinical assessment for patient treatments and enhance risk/benefit-based strategic decisions, we conducted a study using preoperative features with the purpose of developing a risk classifier that anticipates in-hospital death in Chinese patients with ATTAD.

## Methods

### Study design and participants

From 1 January 2016 to 31 December 2021, a single-center, retrospective cohort research was set up. The derivation cohort consisted of 340 consecutive patients with ATAAD who underwent surgery at the Tianjin Chest Hospital (Tianjin, China). To externally validate this model, we used a separate data set of 153 patients with ATAAD (validation cohort) from the First Hospital of Nanjing Medical University (Nanjing, China) between 1 January 2004 and 31 July 2018. The Ethics Committee of the Tianjin Chest Hospital authorized the study with regulatory and ethical permission (2023LW-001). It was also approved by the Ethics Committee of the First Hospital of Nanjing Medical University. Due to the retrospective nature, informed written consent was waived. These individuals were not included if they had a history of trauma, pregnancy, iatrogenic aortic dissection, Marfan syndrome, infections, tumors, or other conditions involving the immune and circulatory systems.

### Candidate predictors

Medical records were used to collect valid information for almost every patient, and under the constraints of available data, all eligible predictors were chosen based on thorough literature studies and clinical findings. Age at operation, weight, sex, and height constituted the continuous and classified baseline data. The clinical profiles included a history of smoking, drinking, hypertension, diabetes, stroke, chronic obstructive pulmonary disease, and coronary heart disease. White blood cell count, neutrophil granulocyte count, monocyte count, lymphocyte count, hemoglobin, platelet count, neutrophil-to-lymphocyte ratio, platelet-to-lymphocyte ratio, monocyte to lymphocyte ratio, n-terminal pro-brain natriuretic peptide, albumin, alanine aminotransferase, aspartate aminotransferase, fibrinogen, hypersensitive c-reactive protein, D-dimer, serum creatinine, estimated glomerular filtration rate, and blood urea nitrogen were all included in the preoperative testing profiles. Intraoperative variables included death, mechanical ventilation assistance time, hospital days, and intensive care unit stay time. Table 1 provides a list of these specific and thorough definitions.

**Table 1:** Candidate predictors of 340 patients with ATAAD in the derivation cohort.

**Candidate predictors**	**Survival (*N* = 261)**	**Death (*N* = 79)**	***P*-value**
Height (cm)	171.10 ± 7.80	169.73 ± 8.74	0.185
Age (year)	53.11 ± 11.65	54.03 ± 12.78	0.549
Weight (kg)	79.33 ± 16.44	81.03 ± 18.56	0.436
Male, *n* (%)	193 (73.95%)	57 (72.15%)	0.189
WBC, 10^9^/L	11.20 ± 3.7	13.42 ± 3.67	<0.001
NEUT, 10^9^/L	9.53 ± 3.77	11.66 ± 3.99	<0.001
LYM, 10^9^/L	1.04 ± 0.56	1.03 ± 0.61	0.867
MONO, 10^12^/L	0.57 ± 0.28	0.63 ± 0.24	0.135
HGB, g/L	131.72 ± 22.24	133.53 ± 17.46	0.506
PLT, 10^9^/L	181.89 ± 66.98	177.89 ± 50.64	0.624
PLR	215.93 ± 114.11	227.61 ± 161.86	0.473
NLR	12.39 ± 8.28	15.21 ± 11.18	0.016
LMR	2.19 ± 1.50	1.78 ± 0.94	0.023
BNP, pg/ml	116.40 (41.32–422.70)	154.40 (61.71–472.9)	0.351
ALB, g/L	40.00 (37.30–43.00)	39.80 (37.73–41.95)	0.587
ALT, U/L	18.60 (12.20–28.80)	21.20 (14.60–34.70)	0.036
AST, U/L	20.10 (15.40–28.80)	21.90 (16.10–48.75)	0.062
FIB, mg/dl	2.67 (2.19–3.35)	2.43 (1.79–3.16)	0.280
D-Dimer, mg/L	9.28 (2.99–20.00)	14.18 (3.61–20.00)	0.117
CRP, mg/L	7.47 (2.41–23.23)	7.19 (2.97–18.30)	0.393
Scr, mmol/L	94.89 ± 42.77	114.68 ± 71.81	0.003
eGFR, ml/min	95.70 ± 34.29	90.29 ± 48.64	0.27
BUN, mg/dL	6.57 ± 2.42	7.73 ± 4.01	0.002
Drinking, *n* (%)	108 (41.38%)	27 (34.18%)	0.252
Smoking, *n* (%)	136 (52.11%)	44 (55.70%)	0.576
Hypertension, *n* (%)	184 (70.50%)	61 (77.22%)	0.244
Diabetes, *n* (%)	15 (5.75%)	2 (2.53%)	0.251
Stroke, *n* (%)	22 (8.43%)	6 (7.59%)	0.813
COPD, *n* (%)	3 (1.15%)	0 (0.00%)	0.338
CHD, *n* (%)	20 (7.66%)	13 (16.46%)	0.021
Arrhythmia, *n* (%)	9 (3.45%)	2 (2.53%)	0.687
MVA times (hour)	52.00 (17.00–119.00)	85.00 (27.50–239.01)	<0.001
Hospital day (day)	15.00 (11.00–20.00)	11.00 (3.00–16.00)	<0.001
ICU stay time (day)	7.00 (4.00–11.00)	7.00 (3.00–14.50)	0.169

WBC, white blood cell count; NEUT, neutrophil granulocyte count; LYM, lymphocyte count; MONO, monocyte count; HGB, hemoglobin; PLT, platelet count; PLR, platelet-to-lymphocyte ratio; NLR, neutrophil-to-lymphocyte ratio; LMR, lymphocyte–monocyte ratio; BNP, n-terminal pro-brain natriuretic peptide; ALB, albumin; ALT, alanine aminotransferase; AST, aspartate aminotransferase; FIB, fibrinogen; CRP, hypersensitive C-reactive protein; Scr, serum creatinine; eGFR, estimated glomerular filtration rate; BUN, blood urea nitrogen; COPD, chronic obstructive pulmonary disease; ICU, intensive care unit; CHD, coronary heart disease; MVA time, mechanical ventilation assistance time.

### Study outcome

In-hospital death served as the major clinical endpoint. Mechanical ventilation assistance time, hospital days, and ICU stay time are the secondary outcomes.

### Statistical analysis

Variables in the derivation and validation cohorts were checked for missing values ahead of data analysis. The percentage of missing data among the predictors ranged from 0 to 31.7%. Using the mice package for R, which embeds predictive mean matching with the cases (k) = 5 default, we imputed incomplete information by multiple imputations using chained equations to include these data throughout the analyses.

The model was carried out in accordance with the recommendations of Transparent Reporting of a Multivariable Prediction Model for Individual Prognosis or Diagnosis (TRIPOD) (10). We added a collection of preset prediction factors for preoperative variables made up of clinical characteristics and data from preoperative testing (Table 1). To choose the most helpful prediction factors from those candidates in the cohorts, we then used the least absolute shrinkage and selection operator (LASSO) analysis in a penalized logistic regression model (R package glmnet) (11). LASSO applies a penalty to variables, ultimately only selecting ones that contribute to the out-of-sample performance by utilizing cross-validation. This results in excellent predictive performance in datasets with potential multi-collinearity from many predictor variables and does not rely on *P*-values. Using the product of the expression levels for the variables chosen by the LASSO analysis and the corresponding regression coefficients weighted by logistic regression analysis in the cohort, we established the prediction scoring model by allocating each patient a risk score for postoperative in-hospital death. Afterward, using generalized additive models, we fitted the correlation between the risk score and in-hospital death. Then, we fitted the relationship between the risk score and in-hospital death using generalized additive models and found the optimal cut-off point using EmpowerStats software (X&Y Solutions). The thresholds for the scores that were output from the predictive model that was used to classify patients into different risk categories were defined as the scores with the highest log-likelihood value in a regression model. We divided patients into low-, middle-, and high-risk categories based on the inflection of the risk score curve with in-hospital death. Multiple comparisons of in-hospital death rates against a control group (low-risk category) were conducted using Dunnett's method. In addition, we compared our new model with the prior published nomogram for acute thoracic dissection, and we found that this risk model is significantly superior to Yang's nomogram (12).

In total, 153 patients with ATAAD from the First Hospital of Nanjing Medical University were used as an independent external data set to evaluate the external validity of model performance. We examined the discrimination ability [area under receiver operating characteristic curve (AUC)] and clinical application ability (decision curves), which assess the net benefit of nomogram-assisted decisions. Using logistic regression for baseline data, we subsequently evaluated the relationship between risk classifications and in-hospital death.

For continuous variables, data are displayed as frequencies (percentages) for categorical variables and medians [interquartile ranges (IQRs)]. The χ^2^ test or Fisher exact test for categorical variables and the Student *t*-test or the Mann–Whitney U-test for continuous variables analyzed group differences. A two-sided *P*-value of 0.05 was regarded as statistically significant. We conducted the statistical analysis using Stata v14 (StataCorp) and R software (v3.2.0; R Foundation for Statistical Computing).

## Results

**Figure 1:**
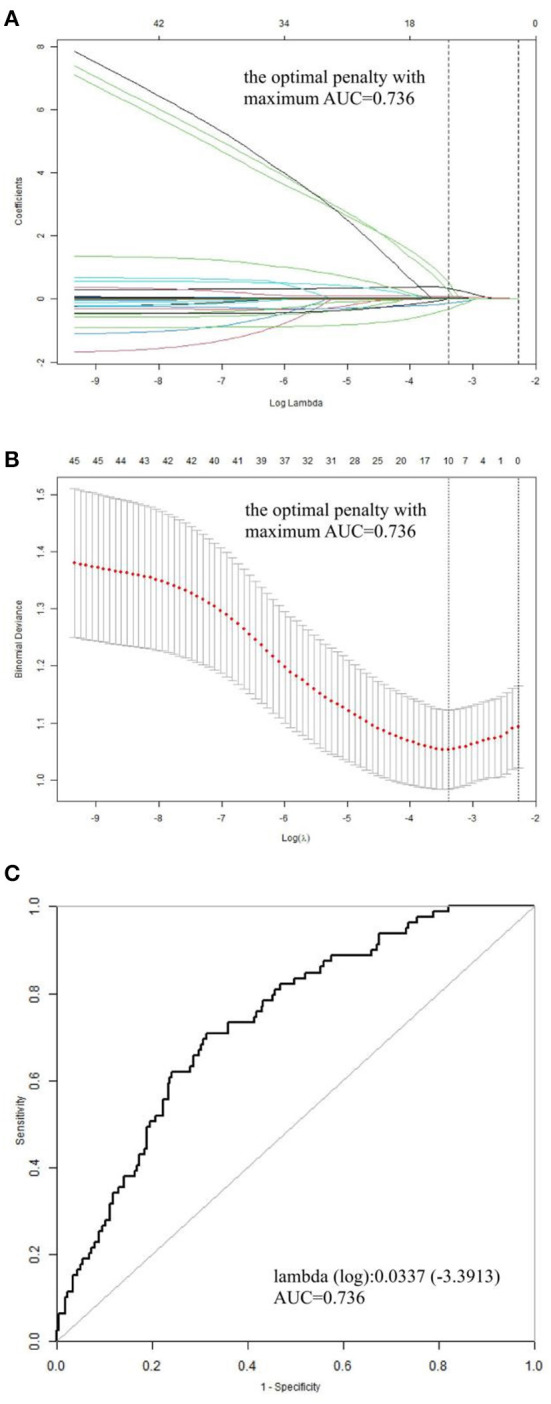
Profile charts for the LASSO model. **(A)** The factors and their regression coefficients were chosen for the model based on the optimal k for the LASSO model, and the coefficient profile graphs demonstrate how the size of the preoperative variables' coefficients keeps shrinking with the growing quantity of the k penalty. **(B)** Plotting penalties for the LASSO model. The standard error is displayed as colored error bars. **(C)** The ideal κ penalty for the LASSO model has an AUC maximum of 0.736. AUC, area under the receiver operating characteristic curve; LASSO, least absolute shrinkage and selection operator.

The derivation cohort comprised 340 patients with ATAAD who had undergone surgical treatment, with a mean age of 53.32 years. Of these patients, 90 were women, constituting 26.47% of the total (Table 1). The validation cohort consisted of 153 patients, with a mean age of 54.67 years and including 39 (46.2%) women (Table 2). The occurrence of in-hospital death was 23.24% (79/340) in the derivation cohort and 15.69% (24/153) in the validation cohort. Baseline clinical characteristics in the cohort are listed in Table 1. In the death group, mechanical ventilation assistance time was much longer than the survival group [median: 85.00 (IQR: 27.50–239.01) vs. 52 (IQR: 17.00–119.00], *P* < 0.001). The hospital day for the death group seemed to be shorter compared to that of the survival group [median: 11.00 (IQR: 3.00–16.00) vs. 15 (IQR: 11.00–20.00), *P* < 0.001]. We later discovered a hybrid panel using the LASSO analysis that included five factors with the optimal k penalty that were related to in-hospital death in the cohort (AUC = 0.736; Table 1, Figure 1). The expression levels of these five factors were weighted by their regression coefficients, and a risk score was calculated for each patient using this procedure: Risk score = 0.00067^*^Scr + 0.01437^*^D-Dimer + 0.07890^*^WBC+ 0.31527^*^CHD + 0.02788^*^BUN. Their predictive importance is shown in Supplementary Figure 1 (Scr: serum creatinine; WBC: white blood cell count; CHD: coronary heart disease; BUN: blood urea nitrogen).

**Table 2:** Patient characteristics and outcomes in derivation and validation cohorts.

	**Derivation cohort (*N* = 340)**	**Validation cohort (*N* = 153)**
In-hospital death	79 (23.24%)	24 (15.69%)
Height (cm)	170.79 ± 8.03	169.42 ± 8.42
Age (year)	53.32 ± 11.91	54.67 ± 12.54
Weight (kg)	79.72 ± 16.94	72.15 ± 12.74
Male, *n* (%)	250 (73.53%)	114 ± 74.51%
WBC, 10^9^/L	11.72 ± 3.86	11.15 ± 4.88
NEUT, 10^9^/L	10.02 ± 3.92	9.12 ± 4.78
LYM, 10^9^/L	1.04 ± 0.57	1.26 ± 0.76
MONO, 10^12^/L	0.59 ± 0.27	0.72 ± 0.39
HGB, g/L	132.14 ± 21.21	134.29 ± 17.99
PLT, 10^9^/L	180.96 ± 63.51	180.16 ± 64.07
PLR	218.64 ± 126.64	178.72 ± 99.12
NLR	13.05 ± 9.10	10.08 ± 8.02
LMR	2.10 ± 1.40	2.17 ± 1.60
BNP, pg/ml	131.40 (51.39–467.55)	486.40 (307.50–927.20)
Albumin, g/L	41.17 ± 24.83	40.13 ± 22.75
ALT, U/L	19.05 (12.60–30.83)	33.66 ± 25.58
AST, U/L	20.40 (15.57–31.45)	49.48 ± 116.12
FIB, mg/dl	2.58 (2.10–3.30)	2.77 ± 1.58
D-Dimer, mg/L	11.37 (3.10–20.00)	6.43 ± 10.88
CRP, mg/L	7.42 (2.51–23.20)	32.84 ± 28.52
Scr, mmol/L	99.49 ± 51.57	92.79 ± 114.34
eGFR, ml/min	94.44 ± 38.10	86.61 ± 35.86
BUN, mg/dL	6.84 ± 2.90	7.30 ± 5.50
Drinking, *n* (%)	135 (39.71%)	59 (38.56%)
Smoking, *n* (%)	180 (52.94%)	55 (35.95%)
Hypertension, *n* (%)	245 (72.06%)	101 (66.01%)
Diabetes, *n* (%)	17 (5.00%)	2 (1.31%)
Stroke, *n* (%)	28 (8.24%)	13 (8.50%)
COPD, *n* (%)	3 (0.88%)	4 (2.61%)
CHD, *n* (%)	33 (9.71%)	15 (9.80%)
Arrhythmia, *n* (%)	11 (3.24%)	1 (0.65%)
MVA times (hour)	57.00 (19.00–133.50)	35.00 (21.00–86.00)
Hospital day (day)	14.00 (10.00–20.00)	21.00 (17.00–29.00)
ICU stay time (day)	7.00 (4.00–12.00)	7.00 (4.00–11.00)

**Figure 2:**
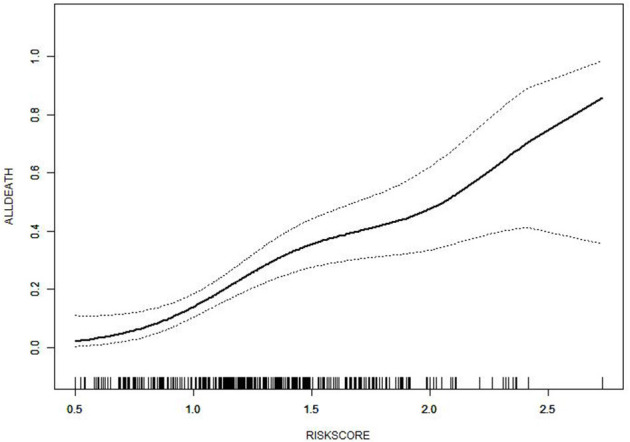
Relationship between risk score and in-hospital death.

**Figure 3:**
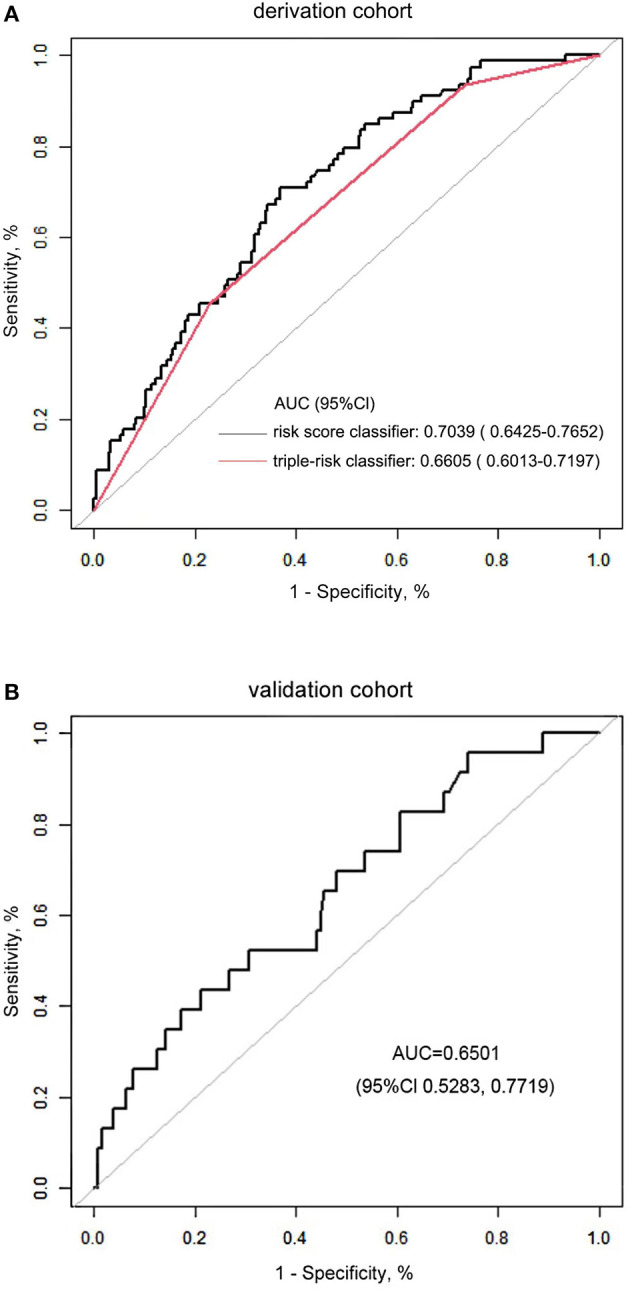
**(A)** Comparison of the risk score classifier and the triple-risk (i.e., low, intermediate, high) classifier. AUC indicates the area under the receiver operating characteristic curve. **(B)** The performance of the risk score classifier in the validation cohort.

The in-hospital risk of death was identified as having an increased risk score. Compared to those who survived, patients who died in the hospital had a substantially higher risk score [median: 1.533 (IQR: 1.238–1.742) vs. 1.237 (IQR: 0.977–1.483), *P* < 0.001; Figure 2]. For the likelihood of hospitalized death in the cohort, patients were divided into three risk categories: low risk (1 or fewer points), intermediate risk (between 1 and 1.5 points), and high risk (1.5 points or more). With reference to the low-risk group with 74 (21.8%) patients, the intermediate group with 170 (50.0%) patients and the high-risk group with 96 (28.2%) patients posed a substantially greater risk of postoperative in-hospital death in the cohort [adjusted OR: 3.973 (95% CI, 1.496–10.552), *P* = 0.00564; 8.280 (95% CI, 3.054–22.448), *P* = 0.00003, respectively]. We performed a comparison of the diagnostic value of the continuous and categorical risk scores. According to the AUC comparison, the risk score classifier exhibited better prediction ability than the triple-risk categories classifier [AUC: 0.7039 (95% CI, 0.6425–0.7652) vs. 0.6605 (95% CI, 0.6013–0.7197); *P* = 0.0022] for predicting in-hospital death. For the risk score classifier, the specificity and sensitivity were 0.6322 and 0.7089, and for the triple-risk categories classifier, the specificity and sensitivity were 0.7701 and 0.4557, respectively (Figure 3A). In order to test the risk score classifier's performance, the discrimination was a little lower in the external validation cohort [AUC: 0.6501 (95% CI, 0.5283–0.7719)], and the specificity and sensitivity were 0.7874 and 0.4348 (Figure 3B).

**Figure 4:**
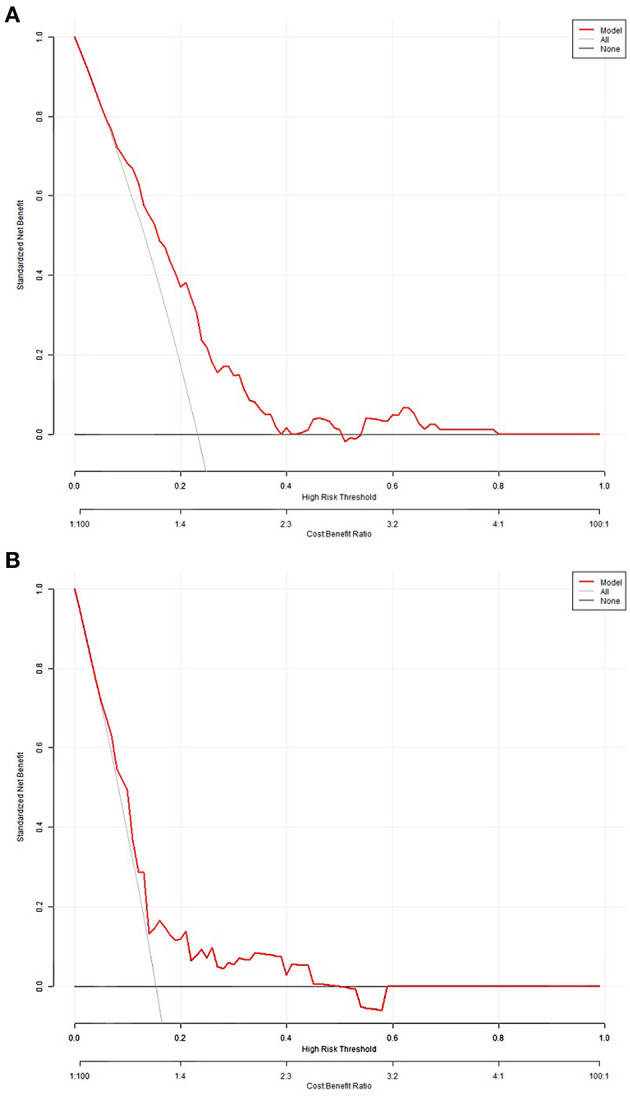
Decision curves for in-hospital death probability in the derivation **(A)** and validation cohorts **(B)**.

The decision curves for in-hospital death probability in the derivation cohort and validation cohort (Figures 4A, B) showed relatively good performance for the model in terms of clinical application. If the threshold probability in the clinical decision was more than 10%, then the use of the risk score classifier to detect in-hospital death showed a greater advantage than assuming that all patients would develop in-hospital death or that no patients would develop in-hospital death.

Consequently, to predict in-hospital death in patients with ATAAD, the risk score classifier was chosen as the major tool instead of the triple-risk categories classifier.

In addition, we compared our new model with the prior published nomogram for patients with ATAAT, and we found that this risk model is significantly superior to Yang's nomogram (Supplementary Table 1).

## Discussion

In this single-center retrospective cohort study, we created a novel predictive algorithm based on five preoperative characteristics chosen to greatly enhance the ability to forecast in-hospital death in Chinese patients with ATAAD. Patients with high-risk ATAAD who underwent surgery may be identified by clinicians using this prediction model. The straightforward application of these fewer variables for early risk-stratification in patients with ATAAD presenting to the emergency department seems to render them appealing, quick, and easy triage tools, especially if their application would result in a quantitatively lower death possibility and better prognoses for patients. The optimized tool showed good discrimination. Our findings demonstrated that patients in the intermediate and high-risk groups exhibited a significantly higher probability of dying in the hospital than those in the low-risk group.

Given that ATAAD is a high-risk disease, progresses rapidly, and has a high mortality rate, it may be possible to develop better predictive and therapeutic methods by investigating the key factors that contribute to and initiate postoperative hospital death. According to the findings of our research, there is a set of five preoperative factors which could accurately forecast in-hospital death in patients with ATAAD. Patients with higher preoperative levels of Scr, BUN, WBC, and D-dimer and a history of CHD had a higher risk of in-hospital death.

D-dimer is a protein fragment produced by crosslinked fibrin which is detectable in plasma after thrombus fibrinolysis and serves as a biomarker for the synthesis of the coagulation-fibrinolysis balance (13). Aortic dissection and subsequent aortic rupture are brought on by thrombosis and inflammation, and D-dimer is a key factor in thrombosis (14, 15). D-dimer levels below a previously defined cut-off reflect a negligible or non-existent thrombus formation, conversely, the appearance of D-dimer in circulating plasma beyond that of a specified level can, indeed, indicate the likelihood of an unnoticed thrombosis, with values typically assumed to be related to clot burden (15, 16). D-dimer has been demonstrated in recent years to be significant for prognostication in a variety of cardiovascular illnesses (17, 18). D-dimer testing has already been advised for the rule-out of ATAAD since earlier research has demonstrated a strong link between increased D-dimer levels and in-hospital death (19–22). As said by Feng et al., increased D-dimer levels were found to be independently related to in-hospital major adverse events and can, therefore, be employed as a helpful predictive biomarker prior to operation with ATAAD (21). In addition, Tang et al. also discovered that elevated D-dimer is an independent predictor of unfavorable in-hospital outcomes in patients with ATAAD (22). According to the findings of this research, patients with ATAAD may have higher D-dimer levels, which is in line with earlier studies and might be a predictive sign of poor results (20–22), despite the fact that the disparity was not statistically significant, perhaps a larger sample size will be required to test it again.

The inflammatory reaction is a significant contributing factor to the progression of aortic dissection. The inflammatory response could be brought on by the aortic tissue injury and thrombus in the fake lumen generated by the dissection. The tissues of the torn aorta have been found to have WBC, including neutrophils and macrophages (23, 24). The perioperative elevated WBC count (a generator of inflammation) was linked to an increased rate of in-hospital death and was served as a kind of risk variable for a composite adverse event involving heart, lung, brain, and systemic condition. However, The special impact of the WBC on the surgical outcome of TAAAD remained unelucidated. It was said that patients with high preoperative WBC had a poor prognosis and responded worse than those with normal WBC (25, 26). The post-discharge mortality in individuals with ATAAD is independently predicted by relatively high WBC on admission, according to Zhang et al. (27). Ke et al. also said that increased WBC can be employed as supplementary markers for postoperative in-hospital death with ATAAD (28). Elevated preoperative WBC was associated with a higher risk of death following an ATAAD procedure in this research, and this could be caused by inflammation from a vascular intima rupture, just as the difference in WBC between the two groups was statistically significant, which is in line with the findings of the outcomes of past experiments (29).

In recent years, it has been demonstrated that preoperative organ malperfusion influences the prognosis for patients with ATAAD (30), and early renal dysfunction before surgery was common in patients with ATAAD (31). The relationship between the prognostic value of preoperative renal dysfunction and postoperative hospital death in patients with ATAAD has not been explored in the literature. Imasaka discovered that there is no clear link between the preoperative estimated glomerular filtration rate and in-hospital mortality in patients with ATAAD (32). However, concerning in-hospital death among patients with ATAAD, Zhou et al. discovered that moderate and severe renal dysfunction were risk factors (33). Fan et al. discovered that preoperatively elevated SCr is associated with death in patients with ATAAD following surgical treatment (34). In patients with type A aortic dissection, Li et al. revealed that increased blood urea nitrogen levels might be a death risk factor (35). Our study enhanced the impact of preoperative renal impairment on postoperative hospital death among patients with ATAAD using the two variables, SCr and BUN, as the conventional reference index for evaluating renal function. The statistical probability of postoperative in-hospital death increases with considerably greater preoperative levels of Scr and BUN, and this difference is statistically significant, this is consistent with earlier research (36).

When treating ATAAD, it is important to take into account the prevalence of coronary artery disease caused by both the included dissection and atherosclerotic stenosis. Nevertheless, it is still unclear what the connection is between ATAAD and coronary artery diseases. Du et al. found that a history of coronary heart disease had a close relationship with AAD and was an independent risk factor for AAD (37). In our study, the history of CHD is associated with postoperative hospital death, and the difference is statistically significant, which is consistent with the results of previous literature (28, 38). However, patients with a history of CHD appeared to be more prevalent in the survival ATAAD group, and we think this may be due to the small sample size.

The limitations of this research should be taken into consideration when evaluating our final results. First, this research is a retrospective single-centered study, which could lead to selection bias. Second, the model does not incorporate enough risk factors. Therefore, more risk factors should be included in the following validation studies to further improve the predictive ability of the model. Third, while the study's operational approach was chosen by weighing up the hazards and advantages of every operation against the available baseline parameters and the inclinations of the cardiologists participating, their specialist skills may not have been the same as those of other practitioners, which could limit the generalizability of these findings in relation to other hospitals. Consequently, in the future, a prospective multicenter large-scale study will always be required to assess the effectiveness of the present findings ahead of their being implemented in the clinical setting.

## Conclusion

In this study, we effectively created a prediction model for in-hospital death in Chinese patients with ATAAD using preoperative indicators. Our results indicate that the risk classifier can also successfully divide patients into various risk categories for in-hospital death, greatly enhancing predictive ability for the evaluation of clinical outcomes. In addition, we successfully demonstrated that the overall risk score classifier that might have been optimized could have significantly improved predictive accuracy for identifying patients who might suffer in-hospital death with ATAAD. The classifier would then assist clinicians in choosing a highly customized treatment strategy for patients with ATAAD. Nevertheless, it is extremely important to keep in mind that the individuals who participated in this research were all subjected to a type of operation that is particularly uncommon in a significant portion of the rest of the globe. In order to validate our conclusion, we anticipate enlarging the sample in subsequent research and performing a prospective cohort study.

## Data availability statement

The raw data supporting the conclusions of this article will be made available by the authors, without undue reservation.

## Ethics statement

The studies involving human participants were reviewed and approved by the Ethics Committee of Tianjin Chest Hospital. The patients/participants provided their written informed consent to participate in this study. Written informed consent was obtained from the individual(s) for the publication of any potentially identifiable images or data included in this article.

## Author contributions

ZG contributed to the conception of the study and revise the manuscript. JC and MQ contributed significantly to the collection and assembly of data. JC and YB performed the data analysis and wrote the manuscript. YB helped perform the analysis with the constructive discussion. HL provided the validation cohort data and helped revise the manuscript. All authors have read and approved the final manuscript.

## Supplementary material

Supplementary Figure 1 Feature of importance in the derivation cohort.

Supplementary Table 1 Compare LASSO risk model with previous nomogram model in derivation cohort.
