# Supplementary figures and images for: Prediction of in-hospital death following acute type A aortic dissection
Source: Front Public Health. 2023 Mar 29;11:1143160. doi: 10.3389/fpubh.2023.1143160 (PMC10090540; doi:10.3389/fpubh.2023.1143160)

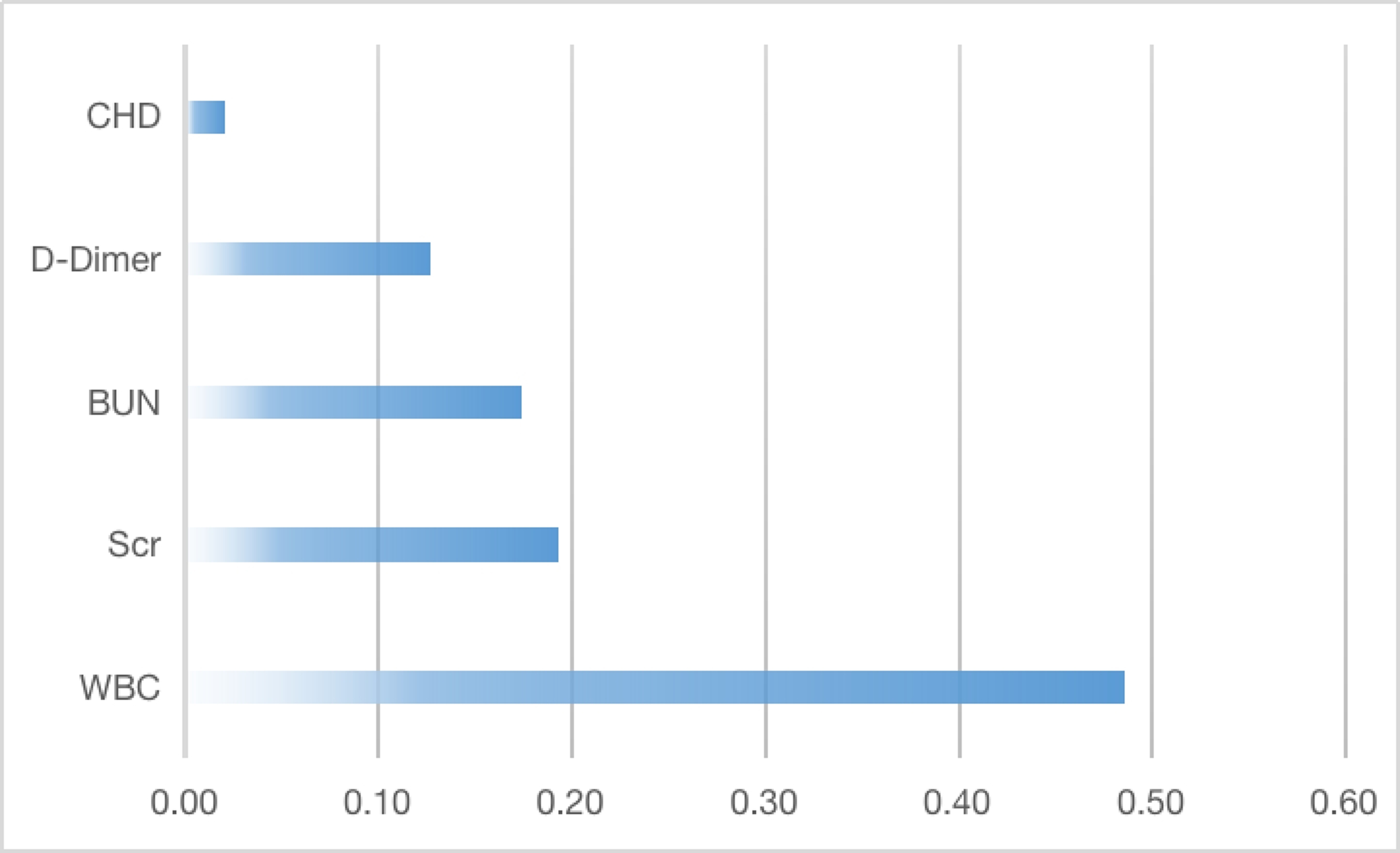

Supplement: Supplementary Figure 1 — Feature of importance in the derivation cohort. [file Image_1.JPEG]
