# Supplementary material for: Prediction of in-hospital death following acute type A aortic dissection
Source: Front Public Health. 2023 Mar 29;11:1143160. doi: 10.3389/fpubh.2023.1143160 (PMC10090540; doi:10.3389/fpubh.2023.1143160)
Supplement: Supplementary Table 1 — Compare LASSO risk model with previous nomogram model in derivation cohort. [file Table_1.docx]

Supplemental Table 1. Compare LASSO risk model with previous nomogram model in derivation cohort

|  | Current LASSO model | Yang’s nomogram |
| --- | --- | --- |
| AUC | 0.7039 | 0.6334 |
| 95%CI | 0.6425-0.7652 | 0.5135-0.7532 |
| Specificity | 0.6322 | 0.8140 |
| Sensitivity | 0.7089 | 0.4167 |
